# Flow Cytometric Analysis of Macrophages and Cytokines Profile in the Bronchoalveolar Lavage Fluid in Patients with Lung Cancer

**DOI:** 10.3390/cancers15215175

**Published:** 2023-10-27

**Authors:** Iwona Kwiecień, Elżbieta Rutkowska, Agata Raniszewska, Agnieszka Rzeszotarska, Małgorzata Polubiec-Kownacka, Joanna Domagała-Kulawik, Jolanta Korsak, Piotr Rzepecki

**Affiliations:** 1 Laboratory of Hematology and Flow Cytometry, Department of Internal Medicine and Hematology, Military Institute of Medicine-National Research Institute, Szaserów 128 Street, 04-141 Warsaw, Poland; erutkowska@wim.mil.pl (E.R.); araniszewska@wim.mil.pl (A.R.); 2 Department of Clinical Transfusion Medicine, Military Institute of Medicine-National Research Institute, Szaserów 128 Street, 04-141 Warsaw, Poland; arzeszotarska@wim.mil.pl (A.R.); jkorsak@wim.mil.pl (J.K.); 3 Department of Surgery, Institute of Tuberculosis and Lung Diseases, Płocka 26 Street, 01-138 Warsaw, Poland; m.polubiec@igichp.edu.pl; 4 Institute of Clinical Sciences, Maria Curie-Sklodowska Medical Academy, 03-411 Warsaw, Poland; domagalakulawik@gmail.com; 5 Department of Internal Medicine and Hematology, Military Institute of Medicine-National Research Institute, Szaserów 128 Street, 04-141 Warsaw, Poland; przepecki@wim.mil.pl

**Keywords:** macrophages, cytokines profile, bronchoalveolar lavage fluid, CD68, CD163, M1, M2, lung cancer microenvironment, cytokines, IL-1RA, IL-6

## Abstract

**Simple Summary:**

Macrophages are an integral part of the tumor microenvironment, playing a role in immunoregulation. We investigated the antigenic and cytokine macrophages profile derived from bronchoalveolar lavage fluid in lung affected by cancer (cBALF) and healthy lung (hBALF) of 36 patients. Macrophages markers: CD206, CD163, CD80, CD86, CD40, Arginase-1, and CD68 were evaluated by flow cytometry. Cytokines (IL-1 RA, IL-6, IL-10, TNF-α, IL-1β, IL-12, IL-23, and TGF-β) profile was analyzed. There was higher median proportion of macrophages in cBALF than in hBALF. The population of macrophages presented immunophenotype: cCD68+^bright^ CD206+^bright^ CD163+^bright^ CD80+ CD86+ CD40+^bright^ CD45+ cArginase+. We observed some trends in the expression of the analyzed antigens in clBALF and hlBLAF. High concentrations of IL-1RA and IL-6 and correlations between pro- and anti-inflammatory cytokines in cBALF and hBALF supernatants were found. We expanded knowledge of the macrophages polarization, their diversity and unique properties based on the antigenic pattern and cytokine profile.

**Abstract:**

Macrophages play an important role in the suppression and activation of immune anti-cancer response, but little is known about dominant macrophage phenotype in the lung cancer environment, evaluated by bronchoalveolar lavage fluid (BALF). The aim of this study was to characterize macrophages in BALF from a lung affected by cancer (cBALF) and a healthy lung (hBALF) of the same patient regarding their individual macrophage polarization and selected cytokines profile. A total of 36 patients with confirmed lung cancer were investigated. Macrophages markers: CD206 CD163 CD80 CD86 CD40 CD45, Arginase-1, and CD68 were evaluated by flow cytometry. Cytokines (IL-1 RA, IL-6, IL-10, TNF-α, IL-1β, IL-12, IL-23, and TGF-β) profile was analyzed. There was higher median proportion of macrophages in Cbalf than in Hbalf. The population of macrophages presented immunophenotype: Ccd68+^bright^ CD206+^bright^ CD163+^bright^ CD80+ CD86+ CD40+^bright^ CD45+ cArginase+. We observed some trends in the expression of the analyzed antigens in clBALF and hlBLAF. The highest concentrations of IL-1RA and IL-6 were in Cbalf and Hbalf supernatant. There were the correlations between pro- and anti-inflammatory cytokines. The findings showed that macrophages include a diverse and plastic group with the presence of different antigens and cytokines, and determining the target phenotype is a complex and variable process.

## 1. Introduction

Lung cancer is the most common malignant tumor and one of the worst prognoses. The median survival time is estimated as less than 5 years after identification [1]. Due to the different clinical picture and therapeutic options, lung cancer is divided into two categories: small cell lung cancer ((SCLC) about 15% of lung cancers) and non-small cell lung cancer ((NSCLC) 85% of lung cancers). NSCLC was divided into lung adenocarcinoma ((ADC) ~40%), squamous cell carcinoma of the lung ((SQCLC) ~25%), and large cell carcinoma (LCC, ~10%) [2]. Despite the emergence of new treatment protocols, the survival rate is low and has one of the highest cancer mortality rates [3]. This is due to the high diversity of the tumor, its insidiousness, low progression-free survival, and resistance to chemotherapy [4].

Currently, tumor immunotherapy is a promising strategy in the treatment of solid tumors. It has become an important achievement for long-term survival in many advanced cases [5]. Modern immunotherapy is designed to improve the antitumor immune defense of the host. Identification of biomarkers helpful in planning this type of individual therapy is another challenge [6]. It turned out that an important prognostic factor is the assessment of the inflammatory infiltration in the tumor mass, including the characteristics of the lymphocyte and macrophage populations as well as the expression of suppressive and regulatory molecules [7].

The tumor microenvironment (TME), poor in nutrients and oxygen, consists of cancerous and non-cancerous cells supporting tumor growth, invasion, and metastasis [8]. In addition, immune cells lose their antitumor capacity and antagonize antitumor activity [9]. The interconversion of tumor-associated macrophages (TAMs), the abundant population in lung cancer, is determined by TME [10].

Macrophages may play an important role in inhibiting the anti-cancer response; however, the direction of macrophage polarization and the nature of the dominant subpopulation at the site of tumor development require clarification [11]. Due to the recent numerous studies on the function of macrophages and the growing number of discovered ligands and receptors, it is necessary to evaluate these cells by subpopulations. At present, there are two main phenotypes of macrophages: M1 and M2 [12]. M1 is a population of macrophages classically activated by stimulation of the Toll-like receptor (TLR) with appropriate molecules such as bacterial lipopolysaccharides (LPS) and some cytokines, mainly interferon gamma (INF-γ) and tumor necrosis factor alfa (TNF-α). M1 exhibits pro-inflammatory activity and produces high concentrations of: IL-1β, IL-6, IL-12, and IL-23, TNF-α [12]. In addition, it has been shown that M1 plays a role in the response associated with Th1 and Th17 lymphocytes, they have an increased ability to eliminate cancer cells [13]. M2-typed macrophages perform immunosuppressive functions, participate in the processes of angiogenesis and tissue remodeling, and play a role in the regulation of the immune response. M2 macrophages also secrete cytokines just like IL-1RA, IL-10, and TGF-β [14]. Due to its proangiogenic and immunosuppressive activity, it has been shown that M2 is involved in promoting the development of cancerous tumors and may be important in weakening its own anti-tumor response in lung cancer [15]. It has also been shown that M2 is capable of inducing the differentiation of regulatory T cells, while regulatory T cells promote polarization towards M2 cells by secreting IL-10 [16]. The population of macrophages can also be differentiated on the basis of the presence of surface and cytoplasmic markers [17], although the selection of characteristic markers is not fully understood. The antigens CD163 and CD206 have been shown to be a marker specific to the M2 population [18]. M1 macrophages are characterized by high expression of the MHC II molecule and CD80/CD86 co-stimulatory molecules, which indicates their ability to present antigen and functionally makes them APC cells [19]. Macrophages show the ability to quickly adapt to changes in the environment, which may result in switching functions and specific surface markers. Due to their extraordinary plasticity, it is difficult to clearly define the phenotype of these cells. They often show intermediate function and antigenic characteristics for both M1 and M2 populations [20].

The results of the work suggest that a thorough study of the polarity of TAMs, which in solid tumors accounts for about 50% of the tumor mass, may be a new therapeutic strategy in cancer treatment [15,21,22]. It should be noted, however, that in lung cancer research, the greatest obstacle is the poor accessibility of the tumor mass. The vast majority of lung cancer cases are not resectable, which indicates searching for another available population of macrophages in the immediate vicinity tumor. Bronchial lavage fluid (BALF) testing is a method of assessing the immune status of lung cancer patients and determining the tumor microenvironment before starting immunotherapy [23,24,25]. Bronchoalveolar lavage (BAL) is a well-established method for obtaining material from the respiratory tract to determine the type of local immune response [26]. The liquid form of BAL allows for cytometric analysis of the obtained material, assessment of the cells immunophenotype and the intensity of expression of surface and cytoplasmic antigens [27]. To the complete immunological picture of the tumor environment can be added the concentration and mutual correlation of cytokines [28]. The BALF procedure is performed during routine diagnosis of bronchofiberoscopy, with little invasiveness for the patient [29,30].

The use of BALF is a novel aspect of this work and an additional goal of grounding the importance of fluid assessment in lung cancer biology research and clinical practice. It creates the possibility of a new direction for the pre-treatment assessment of patients and the development of additional markers prognostic. Using BALF to understand the role of regulatory elements in the lung cancer environment, among which TAM populations play an important role, may have robust therapeutic implications.

The aim of this study was to characterize macrophages in the BALF from lung affected by cancer (cBALF) and healthy lUNG (hBALF) of the same patient regarding their individual macrophage polarization and cytokines profile.

## 2. Materials and Methods

### 2.1. Patients

BALF was collected from 57 patients while performing diagnostic bronchoscopy in the Department of Surgery, National Institute of Tuberculosis and Lung Diseases, Warsaw, Poland. The study was preceded by the signing of an informed consent by each patient (the Military Institute of Medicine Ethics Committee, 47/WIM/2017). Histological examination confirmed primary lung cancer. The following exclusion criteria were used: history of anticancer therapy, clinical signs of inflammation, chronic obstructive pulmonary disease (COPD), autoimmune diseases, and use of immunosuppressive drugs. Ten patients with unconfirmed NSCLC and four patients with metastatic adenocarcinoma were excluded from the study. In addition, patients in whom macroscopic and microscopic assessment of the obtained material was inconclusive were not included in the study.

Upon confirmation of primary NSCLC, the final study group consisted of 36 patients: 18 women and 18 men; average age: 69.6 ± 5.9 years; range (min–max): 53–83 years. (Table 1). Demographic and clinical descriptions of patients with lung cancer and patients excluded from the study are provided in Appendix A, Table A1. The stage of the disease was determined using the eighth classification of malignant carcinomas TNM [31].

The control group in the study consisted of BALF taken from the same patients but from the opposite lung, not affected by the disease (internal control).

### 2.2. Material

BALF material was collected from both the cancerous lUNG (cBALF) and the other “healthy” lUNG (hBALF) during one bronchofiberoscopy procedure for the diagnosis of lung cancer. A total of 100 mL of 0.9% NaCl solution was administered to each lung and the fluid used was immediately removed. The obtained BALF material was developed as recommended [32]. A cell viability test was performed for each sample using trypan blue and 7-AAD reagent. More than 90% of viability has been achieved [32]. There was no control group; taking BALF from a healthy person without any lung disease is impossible for ethical reasons.

### 2.3. Cell Count and Flow Cytometry Analysis

The Sysmex XN Series Hematology Analyzer (Sysmex Co., Kobe, Japan) was used for measured the number of cells in the BALF. Leukocyte and macrophage populations were assessed using a monoclonal antibody panel using the FACS Canto II BD flow cytometer (Becton Dickinson, Franklin Lakes, NJ, USA). The following antibodies were used to detect leukocyte subpopulations: CD3- FITC, CD8- PE, CD45-PerCP-Cy5.5, CD4-APC (BD Multitest^TM^ catalog number: 342417, clone number: SK7, SK1, 2D1, SK3), CD19-pe-Cy7 (catalog number: 341113, clone number: SJ25C1), CD16-APC-H7 (catalog number: 560195, clone number: 3G8), and HLA-DR-V450 (catalog number: 655874, clone number: L243). For macrophages characteristics we used surface and intracellular staining with panel of antibody: CD206-FITC (catalog number: 551135, clone number: 19.2, BD Biosciences, Franklin Lakes, NJ, USA), Arginasa-1-PE (catalog number: 369704, clone number: 14D2C43 BioLegend, San Diego, CA, USA), CD163-PerCP-Cy5.5 (catalog number: 563887, clone number: GHI/61, BD Biosciences), CD68-PE-Cy7 (catalog number: 565595, clone number: Y1/82A, BD Biosciences), CD86-APC (catalog number: 555660, clone number: B70), CD80-APC-H7 (catalog number: 561134, clone number: L307.4, BD Biosciences), CD40-BV421 (catalog number: 563396, clone number: 5C3 BD Biosciences), and CD45-V500 (catalog number: 655873, clone number: 2D1, BD Biosciences). Cells were surface stained with fluorescently labeled surface antibodies for 20 min at room temperature. For intracellular antibodies (CD68-PE-Cy7 and Arginase-1) detection the additional step with IntraStain (Dako, Glostrup, Denmark) for fixation and membrane permeabilization was carried out. After washing, cells were analyzed within 2 h. For each sample, a minimum of 100,000 events were collected. Based on the experience of a previous flow cytometry study [33], Fluorescence Minus One (FMO) control for the characteristics of selected anti-macrophage antibodies and isotype control were used to detect macrophages and to eliminate the high autofluorescence of these cells.

Data were analyzed with DIVA Analysis software 8.0.1 (BD Biosciences), Infinicyt 1.8 Flow Cytometry (Cytognos, Salamanca, Spain) and Kaluza C Flow Cytometry Software Version 1.1.2 (Becman Coulter Life Science, Kraemer Blvd., Brea, CA, USA).

### 2.4. Cytokines Concentration Measurement

Cytokine concentrations in cBALF and hBALF supernatants were measured on a Luminex^®^ 100/200™ System (Luminex Corporation, Austin, TX, USA). For every sample, the following 8 cytokines were analyzed: IL-1 RA, IL-6, IL-10, TNF-α (Magnetic Luminex Performance Assay, Human Cytokine Premixed Kit A, catalog number: FCSTM03-04, R&D System, Minneapolis, MN, USA), IL-1β, IL-12 (Magnetic Luminex Performance Assay, Human HS Cytokine Premixed Kit A, catalog number: FCSTM09-02, R&D System, Minneapolis, MN, USA), IL-23 (Magnetic Luminex Performance Assay, Human HS Cytokine Base Kit B, catalog number: LBHS000, R&D System, Minneapolis, MN, USA), and TGF-β (Magnetic Luminex Performance Assay, TGF-β Base Kit, catalog number: LTGM00, R&D System, Minneapolis, MN, USA).

### 2.5. Statistical Analysis

Statistica 13.0 (TIBCO Software, Palo Alto, CA, USA) was used for statistical analysis along with Diva Analysis software 8.0.1 (BD Biosciences Franklin Lakes, NJ, USA)and Kaluza C version1.1 (Beckman Coulter Life Science, Kraemer Blvd., Brea, CA, USA)for data presentation. The results are expressed as medians with interquartile range (Q1–Q3) and median of GMF of markers on macrophages. The Mann–Whitney test was used to compare groups. A value of *p* < 0.05 was considered statistically significant. Spearman’s correlation coefficient is a statistical measure of the strength of a monotonic relationship between quantitative variables.

## 3. Results

### 3.1. Clinical Characteristic of Study Group

Table 1 presents the characteristics of the clinical picture of the studied patients. Participants and excluded patients are presented in the Appendix A, Table A1. In the majority of patients, non-advanced stage lung cancer was observed (stage I, n = 28). Adenocarcinoma was the most common subtype of cancer (n = 23). The small number of patients in each group did not allow comparisons between groups with different types of cancer or between stages of the disease.

### 3.2. Leukocytes Subpopulation in cBALF and hBALF

**Figure 1:**
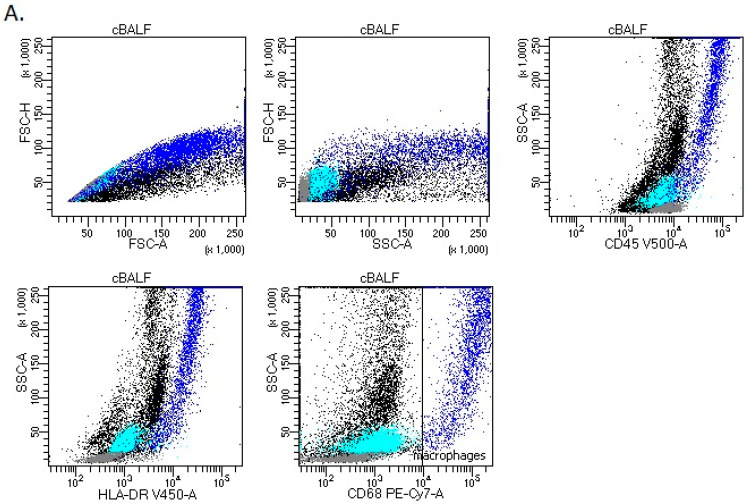

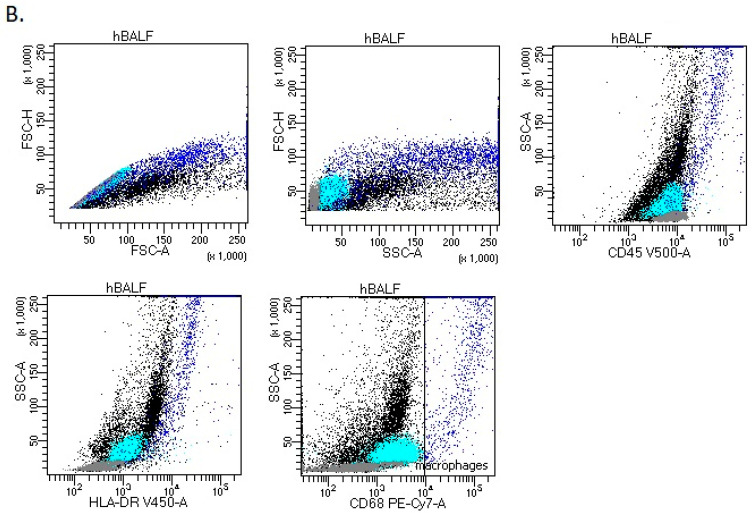
Representative flow cytometry gating analysis of BALF cells ((**A**)-cBALF and (**B**)-hBALF) with antibodies specific for macrophages: FSC-H vs. FSC-A plot: Gating the cells that have an equal area and height, thus removing clumps (greater FSC-A relative to FSC-H and debris (very low FSC). FSC-H vs. SSC-A plot: macrophages (dark blue) are characterized by higher FSC-H and SSC-A. SSC-A vs. CD45 plot: Broad selection of macrophages based on their SSC-A+^high^/CD45+^high^ properties. SSC-A vs. HLA-DR plot: broad selection of macrophages based on their SSC-A+^high^/HLA-DR+^high^ properties. SSC-A vs. CD68 plot: Broad selection of macrophages based on their SSC-A+^high^/CD68+^high^ properties. Other populations: lymphocytes (gray), granulocytes (turquoise). BALF, bronchoalveolar lavage fluid; cBALF, BALF from the lung with cancer; hBALF, BALF from the opposite “healthy” lung.

**Figure 2:**
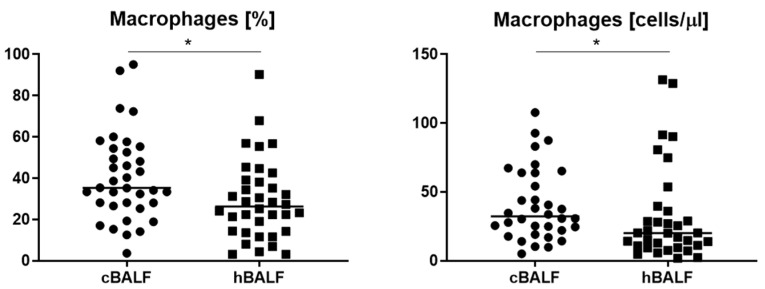
Differences in median proportion and absolute count of macrophages between bronchoalveolar lavage fluid affected by lung cancer (cBALF) and healthy lung (hBALF). The median values (−) was shown on graphs (*, *p* < 0.05).

Firstly, we analyzed leukocytes in cBALF and hBALF. We distinguished the following basic subpopulations of leukocytes: lymphocytes (CD45+^bright^ SSC low), lymphocytes T (CD45+^bright^ SSC low CD3+), CD4 cells (CD45+^bright^ SSC low CD3+ CD4+), CD8 cells (CD45+^bright^ SSC low CD3+ CD8+), lymphocytes B (CD45+^bright^ SSC low CD19+ HLA-DR+), NK cells (CD45+^bright^ SSC low CD16+ CD3-), and neutrophils (CD45+ SSC bright CD16+). We did not notice differences in the leukocyte subpopulation between cBALF and hBALF (Table 2), apart from macrophages. Population of macrophages was analyzed with following immunophenotype: FSC-H+^high^, FSC-A+^high^, SSC-A+^high^, CD45+^high^, HLA-DR+^high^, and CD68^high^ (Figure 1). We observed a higher median proportion and absolute macrophages count in cBALF than in hBALF (Figure 2, Table 2).

### 3.3. Macrophages Phenotype in cBALF and hBALF

**Figure 3:**
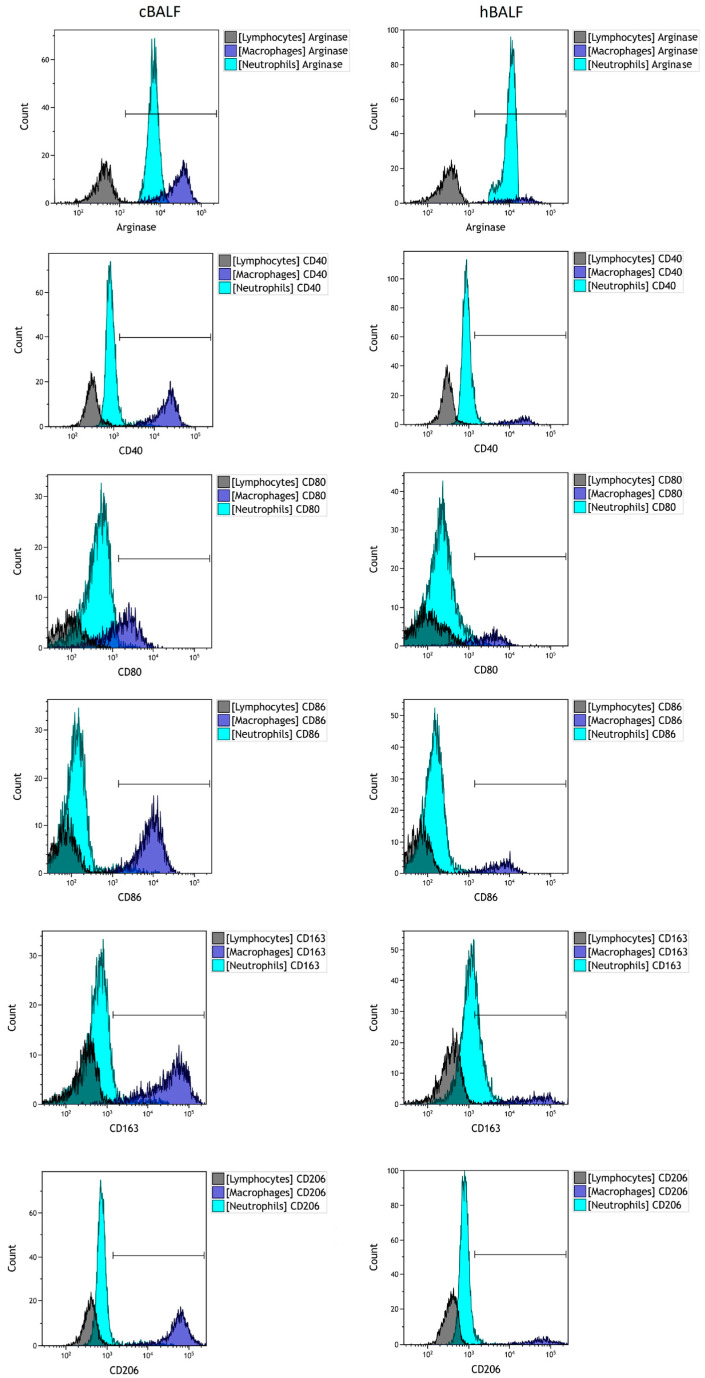
Flow cytometry analysis of selected antigens expression on macrophages, lymphocytes, and neutrophils in cBALF and hBALF from example patient with lung cancer. Histograms show the expression strength of the tested antigens. The marker separates negative from positive cells. BALF: bronchoalveolar lavage fluid; cBALF: BALF from the lung with cancer; hBALF: BALF from the opposite “healthy” lung.

**Figure 4:**
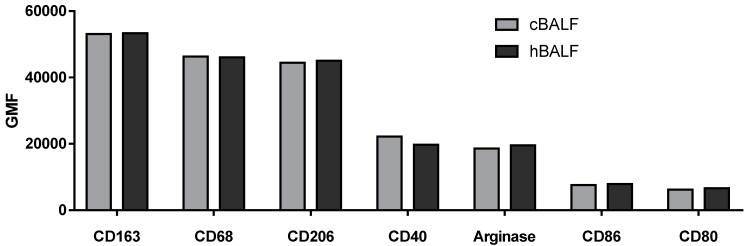
Differences in the geometric mean fluorescence intensity (GMF) of selected antigens between bronchoalveoal lavage fluid from lung affected by cancer (cBALF) and healthy lung (hBALF) and trends in the polarization of macrophages.

Next, we characterized the population of macrophages with the following antibody: CD45+ CD68+ CD206+ CD163+ CD80+ CD86+ CD40+ Arginase+. The expression of given antigens was positive on macrophages in both cBALF and hBALF and oscillated around 100%. The macrophages in both lungs showed the same positive phenotype for selected antigens. Next, we analyzed the geometric mean fluorescence intensity GMF of selected antigens, but there were no statistically significant differences between the GMF of the selected antigens in cBALF and hBALF (Table 3, Figure 3). However, analysis of the GMF of antigens enabled us to evaluate some trends in the polarization of macrophages. The antigens that showed the highest GMF on macrophages were as follows: CD163, CD68, and CD206 (ꜛCD163, ꜛCD68, ꜛCD206). A lower GMF value than for the previously mentioned antigens was observed for: Arginase, CD40, CD86, and CD80 (Table 3, Figure 4).

### 3.4. Cytokines Profile in cBALF and hBALF

The cytokine profile was presented in Table 4. No differences were observed in the concentrations of the cytokines tested: IL-1 RA, IL-6, IL-10, TNF-α, IL-1β, IL-12, IL-23, and TGF-β between cBALF and hBALF supernatants.

**Figure 5:**
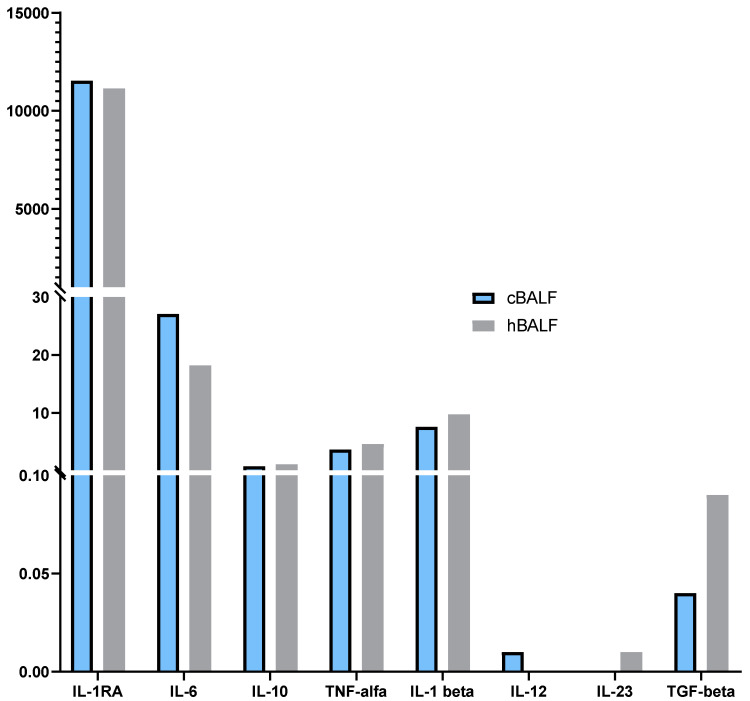
Differences in concentrations of selected cytokines between bronchoalveoal lavage fluid from lung affected by cancer (cBALF) and healthy lung (hBALF) and trends in cytokines profile.

High concentrations of IL-1RA were observed in both cBALF and hBALF supernatants, slightly higher in cBALF supernatants, without statistically significant differences. The high levels of IL-6 and IL-10, TNF-alpha and IL-1b were also determined in cBALF and hBALF (Figure 5).

Considering the correlations between cytokines concentrations, a strong significant positive correlation was observed in cBALF between: IL-1 RA and TNF-α (r = 0.9), for IL-6 and IL-1β (r = 0.5), IL-10 and IL-1 RA (r = 0.9), IL-10 and TNF-α (r = 0.9), IL-10 and IL-1β (r = 0.5), and TNF-α and IL-1 β (r = 0.6), (*p* < 0.05). A weaker relationship, but also a significant one, was found between IL-1 RA and IL-6 (r = 0.3), IL-1 RA and IL-1 β (r = 0.3), TNF-α and IL-12 (r = 0.3), and IL-1 β and IL-12 (r = 0.4) (*p* < 0.05).

The strong significant positive correlation was observed in hBALF between: IL-1 RA and IL-6 (r = 0.6), IL-1 RA and IL-10 (r = 0.9), IL-1 RA and TNF-α (r = 0.9), IL-6 and IL-10 (r = 0.5), IL-6 and TNF-α (r = 0.7), and IL-10 and TNF-α (r = 1.0), (*p*< 0.05). A weaker relationship, but also a significant one, was found between IL-1 RA and IL-6 (r = 0.3), IL-6 and IL-1 β (r = 0.4), IL-6 and IL-12 (r = 0.3), IL-10 and IL-1 β (r = 0.5), and TNF-α and IL-1 β (r = 0.5) (*p*< 0.05).

**Figure 6:**
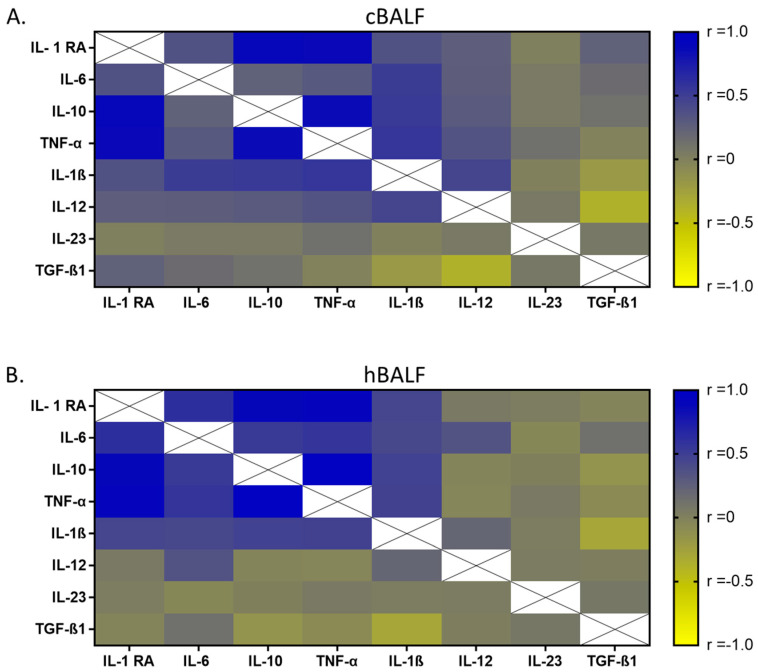
A heatmap of Spearman correlation coefficients for selected cytokines in bronchoalveoal lavage fluid supernatants from lung affected by cancer (cBALF)-(**A**). and healthy lung (hBALF)-(**B**). Correlations with an absolute value more than 0.5 are associated with *p* < 0.05: blue: positive correlations; yellow: negative correlations.

All correlations between the proportion of selected cytokines in cBALF and hBALF supernatants are presented in heat maps (Figure 6).

## 4. Discussion

Macrophages are cells of the innate immune system and are an important component of defense against infections, pathogens, and cancer cells [34]. In the context of lung cancer, macrophages play different roles depending on their state of activity. In some cases, they can help to eliminate cancer cells, while in others, they can promote tumor growth and metastasis [12]. In our work, we evaluated macrophages as part of the regulation of the immune response in lung cancer by analyzing bronchoalveolar lavage (BALF) from cancer-affected lungs (cBALF) as the local environment and healthy symmetrical lungs (hBALF) as a control [35]. Having established the lymphocyte response in TME in lung cancer using BALF in our previous studies [26,35,36], we then undertook to assess the involvement of macrophages. We tested these cells extensively with the assessment of cytokine concentration. Our study confirmed the difficulty of distinguishing M1 and M2 populations, the integrity of the lung environment in relation to the immune response: no significant differences between cBALF and hBALF. We demonstrated the validity of the assessment of the concentration of selected cytokines in the assessment of the local immune response in lung cancer.

### 4.1. Macrophages Count in Bronchoalveolar Lavage Fluid

Bronchoalveolar lavage plays an important role in the diagnosis of interstitial lung diseases, in the differential diagnosis of asthma, chronic obstructive lung disease, and persistent cough and lung cancer. The most numerous group of cells are epithelial cells, a heterogeneous population of follicular macrophages, lymphocytes, neutrophils, and eosinophils [37]. Our previous findings point to the usefulness of BALF analysis in assessing the immune status of a lung cancer patient [35].

In the current work, we found a large population of neutrophils and macrophages in the studied material, and a small number of lymphocytes. We observed higher median proportion of macrophages in cBALF than in hBALF. This observation confirms that macrophages in the vicinity of the tumor are important, it can be concluded that they flow into the disease site, but based on the amount alone we cannot conclude anything about their function in the tumor environment.

Therefore, we decided to immunophenotypically characterize macrophages by assessing the expression of numerous antigens and measured the levels of cytokines concentration that can indicate a dominant population of the macrophage type and the changes in systemic environment.

Distinguishing proinflammatory M1 and immunosuppressive M2 macrophages by phenotype analysis is difficult. In our previous studies, we observed high plasticity of these cells. Macrophages have the ability to adapt quickly to the environment, resulting in switching their functions as their phenotype changes [24,38]. In most tumors studied, macrophages contribute to cancer progression and metastasis through a variety of mechanisms, including promoting cancer cell survival and proliferation, angiogenesis, and suppression of innate and adaptive immune responses [39,40].

Research on examining the sheer amount of macrophages in BALF from cancer patients has yielded conflicting results. Some studies have found that increased numbers of macrophages in BALF are associated with better outcomes in lung cancer patients, while others have found no significant association or even a negative correlation.

Chen L. et.al have shown that infiltrating CD163-positive macrophages are the predominant population in BALF from lung cancer patients. They observed elevated levels of inflammatory factors and more CD163-positive macrophages in lung cancer compared with benign ones, which reflects the local environmental status of host immunity and may be helpful in the diagnosis of lung cancer [41].

Other research, found that high numbers of macrophages in BALF were associated with better survival in patients with non-small cell lung cancer. However, another study found no significant association between macrophage counts in BALF and survival in patients with small cell lung cancer [42,43].

### 4.2. Usefulness of Macrophage Phenotype Assessment

Scientists are investigating the possibility of using macrophages phenotype to predict a forecast of patients with lung cancer. There are no studies that look at the affected lung and the tumor-free lung of the same patient. This is an innovative element of this study.

Our results showed that AMs simultaneously expressed M1-type markers and M2-type markers, while the M2 markers CD163 and CD206 are dominated. Macrophages are a highly plastic population of cells, which may be why it is difficult to identify one dominant phenotype.

Li, J. et al. analyzed alveolar macrophages (AM) in the microenvironment of NSCLC patients. BALF was performed on 20 patients and showed that the AMs simultaneously expressed M1-type markers and M2-type markers, while M2 markers predominated [44]. Macrophages show high phenotypical plasticity caused by specific conditions of the microenvironment in which they are located. After receiving specific environmental stimuli, they polarize and change the expression of their surface markers, and change effector functions [45,46,47].

One of macrophages markers is CD163 protein, which is expressed on macrophages that have an anti -inflammatory phenotype. Studies have shown that the high level of positive macrophages in terms of CD163 in BALF is associated with poor survival in patients with lung cancer [48].

Other researchers have characterized M2 macrophages and highlighted their role as a poor prognostic factor in lung cancer patients. Zhang et al. [49] evaluated cancer-associated macrophages in adenocarcinoma by immunofluorescence assay. M1 macrophages were determined by the presence of CD68+ iNOS+ antigens, while M2 macrophages were determined by the presence of CD68+ CD206+ molecules. Polarization towards the M2 phenotype was prevalent and, together with significant lymphoangiogenesis and emerging metastatic nodes in cancer, was associated with a worse prognosis.

Therefore, it is becoming increasingly clear that M1 and M2 macrophages are characterized by the presence of “intermediate” phenotypes involved in immunoregulation, tissue repair or tumor development and defined by various metabolic pathways, surface markers, and cytokines produced [50]. In addition, the dynamic polarization of macrophages from M1 to M2 involves the presence of intermediate polarity stages distinguished by the expression of specific surface markers.

### 4.3. Cytokines Profile Correleted with Macrophages Phenotype

Changes in cytokine levels in BALF reflect immune responses in lung diseases. Few studies have been conducted to investigate the cytokine concentration of lung cancer in BALF. However, the cytokine profile assessed in BALF shows promising potential to facilitate the diagnosis and understanding of the pathophysiology of lung diseases [51].

M1 macrophages are responsible for production of proinflammatory cytokines, such as IL-1, IL-6, IL-12, and TNF-α, while M2 macrophages stimulate humoral response, tissue remodeling and angiogenesis through the production of anti-inflammatory cytokines (IL-10, TGF-β) and inhibit IL-1 by expression of IL-1RII, as well as IL-1 receptor antagonist (IL-1RA). M2 macrophages have the ability to penetrate into the interior of most cancerous tumors [52]. Macrophages are recruited and could promote tumorigenesis through signals produced by cancer cells and polarized adaptive immune responses. Molecules involved in TAM recruitment and education include TGF-β and cytokines such as IL-4 and IL-1 [53].

In our study, we evaluated the levels of interleukins: 1 receptor antagonist (IL-1 RA), 6 (IL-6), 10 (IL-10), 1 β (IL-1 β), 12 (IL-12), 23 (IL-23), tumor necrosis factor (TNF-α), and transforming growth factor (TGF-β). There were no significant differences in the levels of selected cytokines between the lungs tested. The highest values were achieved for IL-1 RA and IL-6 in both hBALF and cBALF. We showed a significant amount of correlations between pro and anti-inflammatory cytokines, although the most significant were in the case of the level of IL-1 RA and IL-10 and TNF-alpha with no difference between lungs. Other published studies have suggested TGF-β1, interleukin IL-6, and TNF-α as possible diagnostic biomarkers for lung cancer due to their higher serum concentrations in patients with lung cancer [54,55]. Chen Z. et al. [56] suggested that TGF-β1 in BALF may be a valuable biomarker for lung cancer, but the measurement of IL-6 or TNF-α in BALF has poor diagnostic value in lung cancer.

Noteworthy is the high concentration of IL-1 RA in the measured material in both hBALF and cBALF. IL-1 RA seems interesting as a member of the IL-1 family that binds to IL-1 receptors suppressing inflammation [57]. IL-1, as a one of the major proinflammatory cytokines, is upregulated in many cancers and plays a role in inducing immunosuppression in TME [58]. IL-1 RA, as a naturally occurring antagonist of IL-1α and IL-1β signaling, plays a key role in inhibiting IL-1α and IL-1β-induced inflammation [59].

In our work, we deliberately used bronchoalveolar lavage fluid collected from the site of immediate disease (cBALF) and the healthy lung (hBALF) of the same patient, but we conducted the study in these two separate compartments. Presenting our results in relation to BALF material from completely healthy people would be valuable, but, as we mentioned earlier, for ethical reasons at this moment and at the time of designing the study, it was not possible in Polish conditions.

When analyzing macrophages by flow cytometry, methodological limitations should be taken into account. A significant obstacle is autofluorescence. However, the use of additional markers to evaluate the macrophage phenotype allowed for circumvention this problem to some extent. Our goal was to compare the cancer environment with a “healthy” lung. We used the same method for cBALF and hBALF; therefore, a comparative study was possible. Similar limitations apply to the assessment of cytokine levels. Concentrations are highly variable, large scatter is observed and the results should be treated somewhat indicatively. All the more valuable are the observations of significant correlations.

By examining only the concentration of cytokines, it is not possible to determine the pro- or anti-inflammatory profile, but the visible strong correlation between cytokines with different pro- and anti-inflammatory roles indicates the relationship of cytokines of opposite nature and their mutual dependent secretion. The plasticity of macrophages resulting from the phenotype and cytokine profile helps them adapt to the microenvironment by changing the activation state in the M1 or M2 projection.

## 5. Conclusions

Macrophages, which can often be found in normal and inflammatory bronchoalveolar airways, require phenotypic studies and evaluation of the production of growth factors and/or proangiogenic factors in BALF in a cancerous state.

The results discussed here show that macrophages comprise a diverse and malleable group with the presence of different antigens together, a non-uniform cytokine profile combining pro- and anti-inflammatory states.

## Figures and Tables

**Table 1:** Demographics of patients with lung cancer and those excluded from the study.

	n	Age (Years) Mean ± SD	Sex m/f	Stage I/II/III/IV	Subtypes AD/SQCC/LCC/AS
Cancer	36	69.6 ± 5.9	18/18	28/4/4/0	23/9/3/1
X no cancer cells	11	70.7 ± 8.9	7/4	-	-
X metastatic cells	4	60.5 ± 10.8	2/2	-	-
X no continuity of diagnosis	4	62.0 ± 13.9	1/3	-	-
X other diagnosis	2	65.0 ± 5.5	1/1	-	-

Abbreviations: n: number; SD: standard deviation; m: male; f: female; AD: lung adenocarcinoma; SQCC: squamous cell lung carcinoma; LCC: large cell neuroendocrine carcinoma; AS: adenosquamous cell lung carcinoma.

**Table 2:** Median proportion of leukocytes subpopulation in bronchoalveolar lavage fluid (BALF): lymphocytes, lymphocytes T (CD4+, CD8+), natural killer cells, granulocytes, and macrophages in the tumor environment (cBALF) and BALF from healthy lung (hBALF). Data expressed as median (Q1–Q3). (* *p*< 0.05 Mann–Whitney U test).

	cBALF Median (Q1–Q3)	hBALF Median (Q1–Q3)	* *p* < 0.05 Mann–Whitney U Test)
Lymhocytes [%]	10.5 (7.0–17.3)	13.7 (7.6–20.3)	0.310101
Lymhocytes [cells/µL]	9.3 (4.2–15.8)	10.4 (5.1–21.7)	0.373551
Lymphocytes T CD3+ [%]	6.7 (3.5–12.0)	10.6 (5.3–16.2)	0.070508
Lymphocytes T CD3+ [cells/µL]	5.8 (2.5–10.0)	6.8 (3.3–12.6)	0.170413
Lymphocytes T CD3+ CD4+ [%]	1.8 (1.1–3.4)	2.7 (1.3–4.8)	0.142350
Lymphocytes T CD3+ CD4+ [cells/µL]	1.3 (0.8–3.2)	2.1 (0.9–4.7)	0.224691
Lymphocytes T CD3+ CD8+ [%]	3.9 (2.3–7.3)	5.8 (2.9–11.0)	0.183149
Lymphocytes T CD3+ CD8+ [cells/µL]	3.0 (1.7–6.3)	0.1 (0.0–0.4)	0.318384
Ratio CD4/CD8	0.4 (0.2–1.1)	0.6 (0.2–1.1)	0.959834
Lymphocytes B CD19+ [%]	0.2 (0.0–0.4)	0.1 (0.0–0.3)	0.809831
Lymphocytes B CD19+ [cells/µL]	0.1 (0.0–0.3)	0.1 (0.0–0.4)	0.806871
Natural killer (NK) cells [%]	0.3 (0.0–1.5)	0.4 (0.0–2.1)	0.337706
Natural killer (NK) cells [cells/µL]	0.2 (0.0–1.6)	0.6 (0.0–1.4)	0.844019
Neutrophils [%]	44.4 (32.9–61.4)	54.3 (45.6–70.4)	0.070508
Neutrophils [cells/µL]	32.7 (17.6–66.3)	45.1 (26.7–83.9)	0.354547
Macrophages [%]	35.3 (27.3–53.5)	26.3 (14.3–38.6)	* 0.013236
Macrophages [cells/µL]	32.2 (22.1–63.9)	20.1 (10.8–36.1)	* 0.037162

**Table 3:** Differences of antigen expression read as % and GMF intensity. Macrophages in the on macrophages from tumor environment (cBALF) and from healthy lung (hBALF). Data expressed as median (Q1–Q3). (* *p* < 0.05 Mann–Whitney U test).

	cBALF Median (Q1–Q3)	hBALF Median (Q1–Q3)	* *p* < 0.05 Mann–Whitney U Test)
% CD206	97.1 (92.4–98.6)	97.7 (90.2–99.4)	0.888623
% Arginase	98.1 (95.9–99.2)	98.2 (95.9–99.3)	0.888623
% CD163	96.3 (93.1–98.9)	96.6 (92.6–99.3)	0.934892
% CD68	100.0 (100.0–100.0)	100.0 (100.0–100.0)	0.842726
% CD86	96.8 (91.6–99.5)	98.0 (91.7–99.6)	0.648868
% CD80	87.4 (78.5–94.1)	87.4 (74.3–93.2)	0.440583
% CD40	98.8 (95.4–99.7)	98.9 (95.9–99.8)	0.879409
GMF CD206	44,438 (28,803–66,189)	45,054 (28,663–67,334)	0.972072
GMF Arginase	18,646 (12,110–31,417)	19,572 (10,700–38,081)	0.717447
GMF CD163	53,134 (34,389–72,585)	53,340 (26,831–84,638)	0.925616
GMF CD68	46,344 (34,389–71,585)	46,080 (30,582–77,574)	0.916350
GMF CD86	7564 (5070–12,816)	7874 (5896–12,332)	0.861031
GMF CD80	6175 (4084–10,921)	6631 (3268–10,683)	0.833605
GMF CD40	22,213 (11,152–28,548)	19,702 (9424–31,888)	0.761523

**Table 4:** Cytokine concentration in the tumor environment (cBALF supernatants) and BALF from healthy lung (hBALF supernatants). Data expressed as median (Q1–Q3). (* *p* < 0.05 Mann–Whitney U test).

Cytokines [pg/mL]	cBALF Median (Q1–Q3)	hBALF Median (Q1–Q3)	* *p* < 0.05 Mann–Whitney U Test)
IL-1 RA	11,535.57 (6294.640–15,332.25)	11,130.85 (7791.798–14,822.15)	0.977679
IL-6	27.09 (11.934–42.04)	18.19 (9.569–37.08)	0.299483
IL-10	0.80 (0.531–1.34)	1.12 (0.584–1.45)	0.366820
TNF-α	3.69 (1.89–6.26)	4.63 (2.159–6.13)	0.835963
IL-1β	7.60 (3.00–15.80)	9.76 (2.255–18.86)	0.871082
IL-12	0.01 (0.004–0.01)	0.00 (0.004–0.01)	0.085922
IL-23	0.00 (0.00–0.01)	0.01 (0.00–0.01)	0.715991
TGF-β	0.04 (0.00–0.48)	0.09 (0.00–0.27)	0.758215

## Data Availability

The data presented in this study are available in this article.

## Appendix A

**Table A1:** The characteristics of the investigated group.

Case	Sex	Aga	Lung Segments	Diagnosis	Histological Type	Stage	TNM
1	m	69	B1-2L	NSCLC	adenocarcinoma	IB	pT2aN0
2	m	73	B6L	no cancer cells	-	-	-
3	m	67	B6L	NSCLC	squamous cell carcinoma	IB	pT2aN0
4	f	70	B8L	NSCLC	adenocarcinoma	IB	pT2aN0M0
5	m	57	B1R	NSCLC	Large cell neuroendocrine carcinoma	IIIA	pT4N0
6	m	84	B3R	no cancer cells	*-*	*-*	*-*
7	m	69	B6L	NSCLC	adenocarcinoma	IA3	pT1cN0
8	f	73	B5L	NSCLC	squamous cell carcinoma	IA1	pT1aN0Mx
9	f	53	B1-2L i B3L	NSCLC	adenocarcinoma	IIIA	pT1cN2
10	m	62	B6L	metastatic cells	*-*	-	-
11	f	61	B8L	no cancer cells	*-*	-	-
12	f	67	B10L	NSCLC	squamous cell carcinoma	IIIB	pT4N2
13	f	62	B6L	metastatic cells	*-*	-	-
14	f	72	B8,10R	no cancer cells	*-*	-	-
15	f	63	B5L	no continuity of diagnosis	-	-	-
16	m	80	B6L	no cancer cells	-	-	-
17	m	72	B2R	metastatic cells	*-*	-	-
18	f	66	B6R	NSCLC	Large cell neuroendocrine carcinoma	IIB	pT2aN1
19	f	72	B1R	NSCLC	adenocarcinoma	IIIA	pT1aN2
20	f	68	B6L	NSCLC	adenocarcinoma	IIB	pT2aN1
21	f	69	B6L	NSCLC	adenocarcinoma	IB	pT2aN0Mx
22	f	72	B5L	no continuity of diagnosis	-	-	-
23	f	73	B9L/B8L	NSCLC	adenocarcinoma	IB	pT2aN0
24	m	83	B1-2L	NSCLC	adenocarcinoma	IB	pT2aN0M0
25	m	69	B3R	NSCLC	adenocarcinoma-squamous cell carcinoma	IB	pT2aN0Mx
26	f	46	B10R	metastatic cells	-	-	-
27	m	66	B3L	no cancer cells	-	-	-
28	m	80	B6R	NSCLC	adenocarcinoma	IA3	pT1cN0
29	m	65	B9L	NSCLC	squamous cell carcinoma	IB	pT2aN0
30	f	73	B3R	NSCLC	squamous cell carcinoma	IB	pT2aN0
31	m	66	B3R	NSCLC	adenocarcinoma	IA2	pT1bN0
32	f	77	B3R	NSCLC	adenocarcinoma	IB	pT2aN0
33	m	62	B6R	carcinoid	-	-	-
34	m	81	B3R	no cancer cells	-	-	-
35	f	63	B2R	NSCLC	adenocarcinoma	IA2	pT1bN0
36	f	73	B3L	NSCLC	adenocarcinoma	IB	pT2aN0
37	f	70	B3L	NSCLC	adenocarcinoma	IA2	pT1bN0
38	f	71	B2R	NSCLC	squamous cell carcinoma	IA3	PT1cN0M0
39	m	66	B3R	NSCLC	squamous cell carcinoma	IA2	pT1bN0
40	m	67	B2R	NSCLC	adenocarcinoma	IB	pT2aN0
41	m	72	B1R	NSCLC	adenocarcinoma	IB	pT2aN0M0
42	m	69	B9L	NSCLC	Large cell neuroendocrine carcinoma	IA3	pT1cN0Mx
43	f	72	B5L	NSCLC	squamous cell carcinoma	IIB	pT2aN1Mx
44	m	42	B6L	no continuity of diagnosis	-	-	-
45	f	62	B3R	NSCLC	adenocarcinoma	IB	pT2aN0
46	m	71	B6L	NSCLC	squamous cell carcinoma	IIA	pT2bN0Mx
47	f	82	B3L	NSCLC	adenocarcinoma	IA2	pT1bN0Mx
48	m	71	B6R	NSCLC	adenocarcinoma	IA3	pT1cN0
49	m	66	B1R	NSCLC	adenocarcinoma	IB	pT2aN0
50	f	54	B6 8 9 L	no cancer cells	-	-	-
51	m	62	B6L	no cancer cells	-	-	-
52	f	68	B3R	hamartoma	-	-	-
53	f	71	B3R	no continuity of diagnosis	-	-	-
54	m	71	B3R	no cancer cells	-	-	-
55	m	72	B9 B10R	NSCLC	adenocarcinoma	IB	pT2aN0
56	m	73	B1R	NSCLC	adenocarcinoma	IA3	pT1cN0Mx
57	m	74	B9 B10L	no cancer cells	-	-	-

Abbreviation: f: female; m: male; NSCLC: non-small-cell lung cancer.
